# Flapping and powering characteristics of a flexible piezoelectric nanogenerator at Reynolds number range simulating ocean current

**DOI:** 10.1038/s41598-022-20836-x

**Published:** 2022-10-01

**Authors:** Joonkyeong Moon, Giho Kang, Busi Im, Jihoon Kim, Dae-Hyun Cho, Doyoung Byun

**Affiliations:** 1 Department of Mechanical Engineering, Sungkyunkwan University, Suwon, 16419 Republic of Korea; 2 Coastal & Ocean Engineering Division, Korea Institute of Ocean Science and Technology, 385 Haeyang-ro, Busan Metropolitan City, 49111 Korea; 3 Department of Mechatronics Engineering, Gyeongsang National University, Jinju, 52725 Republic of Korea; 4 Department of Energy System Engineering, Gyeongsang National University, 33 Dongjin-ro, Jinju, Gyeongnam 52828 Republic of Korea

**Keywords:** Energy science and technology, Nanoscience and technology

## Abstract

For effective ocean energy harvesting, it is necessary to understand the coupled motion of the piezoelectric nanogenerator (PENG) and ocean currents. Herein, we experimentally investigate power performance of the PENG in the perspective of the fluid–structure interaction considering ocean conditions with the Reynolds number (*Re*) values ranging from 1 to 141,489. A piezoelectric polyvinylidene fluoride micromesh was constructed via electrohydrodynamic (EHD) jet printing technique to produce the β-phase dominantly that is desirable for powering performance. Water channel was set to generate water flow to vibrate the flexible PENG. By plotting the *Re* values as a function of nondimensional bending rigidity (*K*_*B*_) and the structure-to-fluid mass ratio (M*), we could find neutral curves dividing the stable and flapping regimes. Analyzing the flow velocities between the vortex and surroundings via a particle image velocimetry, the larger displacement of the PENG in the chaotic flapping regime than that in the flapping regime was attributed to the sharp pressure gradient. By correlating M*, *Re*, *K*_*B*_, and the PENG performance, we conclude that there is critical *K*_*B*_ that generate chaotic flapping motion for effective powering. We believe this study contributes to the establishment of a design methodology for the flexible PENG harvesting of ocean currents.

## Introduction

Researchers have attempted to replace fossil fuel consumption by harvesting perpetual energy. Nature provides perpetual energy sources in multiple forms, such as ocean currents, tides, wind, and sunlight. According to the International Energy Agency, the total global power generated by tides and marine currents is estimated to be up to 1100 TWh^1^. In particular, ocean energy has advantages over solar and wind energy owing to its reliable and continuous energy supply capability. To convert mechanical energy driven by ocean currents, researchers have regarded the piezoelectric effect as a suitable mechanism for harvesting ocean currents because it can be utilized without intricate components, such as a magnetic field, contract-separation layer, and separate voltage source^2^. Applying a strain to a piezoelectric material or structure increases the electric potential difference between the stretched and compressed components due to the relative movement between the cations and anions in the crystals. Accordingly, the simple underlying mechanism of the piezoelectric effect allows us to conveniently design an energy harvesting device, namely the piezoelectric nanogenerator (PENG), without technical difficulty.

The fluid flow induces the flapping motion of a flexible PENG, generating electricity. Accordingly, to generate more electricity, understanding the coupled motion of the elastic structure and fluid flow has been a longstanding topic, namely the study of fluid–structure interaction (FSI). In the field of PENG engineering, the dynamics of a flexible sheet, which is the simplest type of PENG structure, have been studied to enhance the flapping motion by vortex-induced vibration (VIV)^3–6^. Vortex shedding occurs near a bluff body with a fluid flow. The high-frequency vibration of flexible PENG sheets owing to vortex shedding is desirable for powering.

Theoretical and numerical approaches have been implemented to investigate the flapping motion of flexible sheets using VIV^7–9^. The Reynolds number (*Re*) and nondimensional bending stiffness (*K*_*B*_) are major contributors to the flapping motion. Additionally, the mass ratio (M*) dominates the flapping frequency^10,11^. Based on theoretical and numerical studies, experimental evaluations of sheet-type PENGs have been conducted simultaneously under various *Re* ranges. Binyet et al. reported that the geometric parameters according to the flow velocity range and longer plates were important for fluid–structure interaction modeling for greater power output at *Re* = 40,000 units or less^4^. Pan et al. proposed a simulation and experiment of a polyvinylidene fluoride (PVDF) beam based on vortex-induced vibration with *Re* values ranging from 2000 to 30,000^12^. In addition, the inverted model analyzed the relationship between the flapping kinematics and energy harvesting performance depending on the bending rigidity^13^ and the inclination angle in the *Re* range of hundreds^14^. Bae et al. demonstrated that the regimes of fluttering behavior, including contact and chaotic modes, were defined in a regime map for different flag lengths and flow velocities^15^. With the *Re* value at approximately 35,058, Du et al. demonstrated that the output power can be enhanced when an energy harvester is driven at its resonant frequency by vortex shedding^16^. Piezoelectric energy harvesting design studies were presented by installing a novel spindle-like and butterfly-like bluff body with the galloping phenomena^17^ or by employing vibration levels that vary according to the difference in the spacing of multiple bluff bodies^18–20^ to increase the output. Experimental studies have shown that the chaotic flapping motion of sheet-type PENGs through the fluid–structure interaction properties and geometrical parameters improve PENG performance. Some proposed VIV-based PENGs from the literature ^4,12,14,18,21–24^ are listed in Table S1.

The aforementioned experimental studies were conducted in the *Re* range below 40,000. Most studies have been conducted with air flow rather than water flow. There is no literature that experimentally reports the flapping motion of a sheet-type PENG over a wide range of *Re* values simulating ocean currents. In this study, a water channel setup was prepared to vibrate the flexible PENG with the *Re* ranging from 6200 to 131,200, which simulated the ocean current. We correlated the *Re* and *K*_*B*_ values with the PENG performance to provide a design criterion for powering.

## Experimental section

### Water channel system

**Figure 1:**
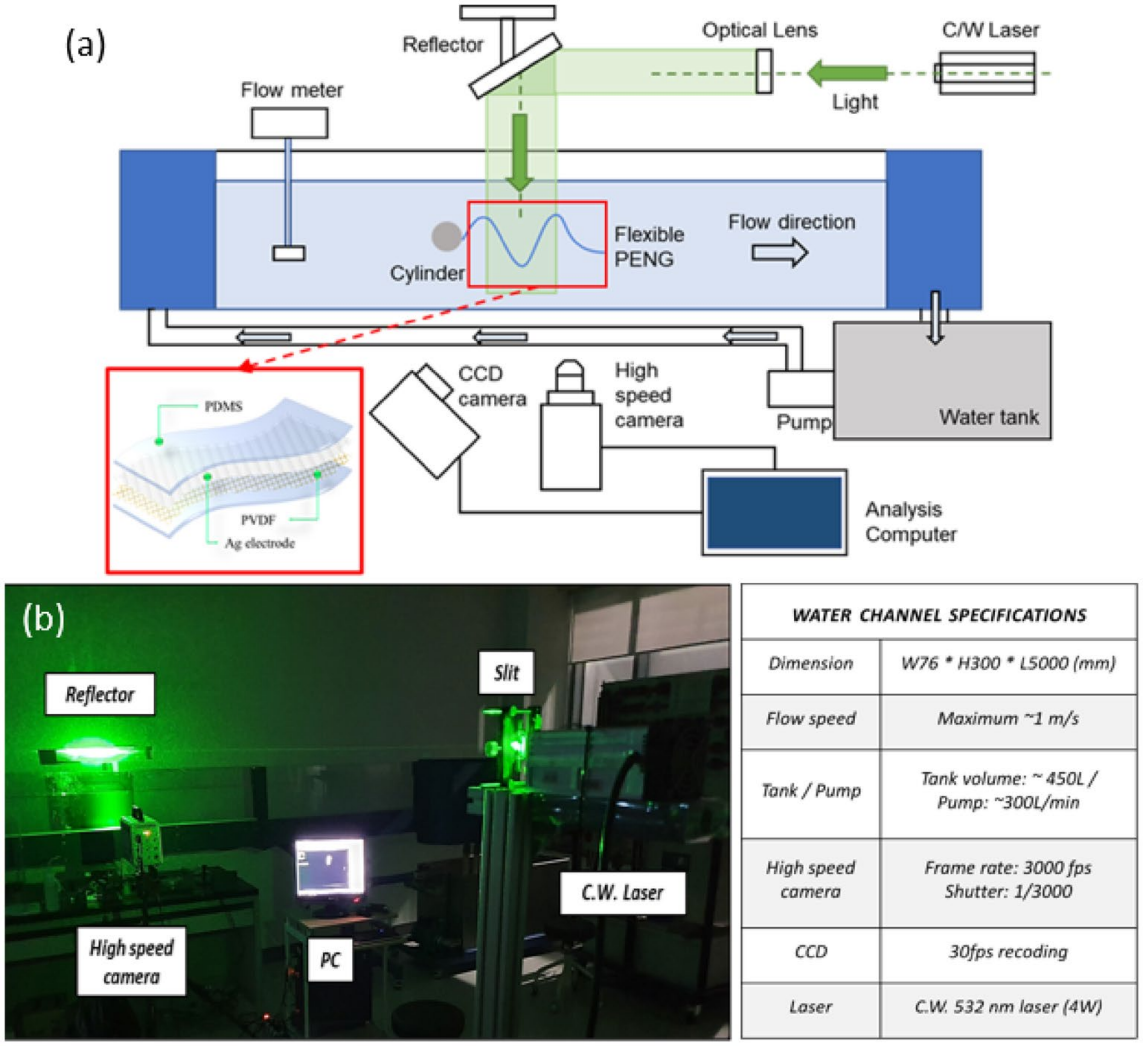
(**a**) Schematic of the water channel system configuration. (**b**) Actual operation image and specifications.

Figure 1 presents a schematic illustration of the overall configuration of the water channel and the flexible sheet-type PENG. The overall length, width, and height of the channel were 5000 mm, 76 mm, and 300 mm, respectively. A water tank pump was used to translocate water to the channel upstream and flow downstream, which made it possible to implement a uniform flow and increase the flow speed to 1 m/s. The flow speed was carefully controlled by adjusting the upper and lower gates and water flux. The flow speed was monitored in real-time using a flow meter on the channel upstream. The PENG was connected in parallel to the flow direction of a circular cylinder located inside a fixed channel. To reduce the disturbances caused by the channel walls and water surface, the PENG was submerged in the center of the water channel. The distance between the PENG and the channel wall is 30 mm, which is much greater than the thickness of the boundary layer thickness (2.37–5.88 mm). The PENG movement was captured using a CCD camera (Firefly FMVU-03MTC, 30fps) and a high-speed camera (Mini ux100, Photron Inc., Japan) installed on the exterior of the channel. A particle image velocimetry (PIV) technique was used to visualize the wake interaction between the structure and fluid flow. Hollow glass microspheres (HGMs) with a diameter of 10 μm (HGM series, Dantec Inc.) were blended with water, and a high-speed camera (Mini ux100, Photron Inc., Japan) was used to observe the movements of the HGMs. Green 532 nm continuous laser (LaserLab, 532 laser 4 W) was passed through the cylindrical and spherical lens to create a thin laser sheet, and irradiated into the channel through a reflector. The laser-irradiated water flow was filmed at 3000 frames per second, and the shutter speed was 1/3000 s; the captured raw data were analyzed using PIV lab software^25^ after preprocessing using the ImageJ program.

### Fabrication of PENG device

#### Preparation of polydimethylsiloxane (PDMS) substrates

The PDMS elastomer base and curing agent (Sylgard184, Dow Corning, USA) mixed in a 10:1 (v/v) ratio was deposited onto a Si wafer by a spin-coating process (Dong-ah trade corp., ACE-200). To construct a 200 μm thickness of the PDMS layer, a rotation speed of 500 rpm was maintained during the spin-coating process, followed by curing in an oven at 150 °C for 30 min.

#### Preparation of PVDF-based printing inks

PVDF (Mw = 534,000) and polyvinylpyrrolidone (PVP) (Mw = 1,300,000) powders were purchased from Sigma-Aldrich. The PVDF and PVP were dissolved in a mixture of N, N-dimethylformamide (DMF) and acetone (1:1 v/v) at 40 °C for 4 h. This blend was used as printing ink. PVDF, which is a highly attractive piezoelectric material owing to its flexibility, ease of fabrication, and chemical stability, was selected to generate electricity. PVP was used to control the viscosity of the blends to obtain a Taylor cone jet at the printing nozzle.

#### Preparation of Ag nanoparticles (AgNPs) based printing inks

The AgNP (10,000 cPs) solution was purchased from NPK Co. (South Korea). The chloroform (≥ 99.8%) and poly (ethylene oxide) (PEO) (Mw ≈ 400,000) were purchased from Sigma-Aldrich (USA). The PEO blended with chloroform was used to adjust the viscosity of the AgNP-based inks. The AgNP solution was mixed with a 10:1 (v/v) PEO solution to obtain a stable Taylor cone jet.

#### Electrohydrodynamic (EHD) jet printing

An electrohydrodynamic (EHD) jet printing technique was employed to construct a 10 μm PVDF fiber mesh pattern on a spin-coated polydimethylsiloxane (PDMS) substrate. An EHD jet printer manufactured by NP-Expert (Enjet Inc., South Korea) with a metal nozzle (NanoNC) was used. To stably eject the Taylor cone jet, a flow rate of 700 nL/min was maintained using a micro syringe pump (Harvard, New PHD UltraTM Nanonomite). The working distance and applied voltage values between the printing nozzle and PDMS substrate were 2200 μm and ranged from 1.6 to 2 kV, respectively. The printing speed was set to 300 mm/s.

#### Fabrication of PENG

Figure S1(a) presents a schematic illustration of the PENG fabrication process. A piezoelectric PVDF-based mesh was constructed using the EHD jet printing technique on the flexible PDMS layer. The AgNP mesh electrodes were printed with a mismatch of a rotation angle of 45° with the PVDF mesh. This mismatch was inevitable when minimizing the overlapping area. The Taylor cone jet of the AgNP ink became unstable when propelled directly onto the PVDF. After printing the AgNP mesh electrodes, a conductive wire was connected to the edge for a power measurement. Finally, the PDMS layer was deposited on top to block the water. Optical images of the fabricated PENG are displayed in Fig. S1(b). The output of the PENG was measured using a picoammeter (6485/E, Keithley Instruments Inc., USA) and electrometer (6514/E, Keithley Instruments Inc., USA). The topographical profiles of the printed AgNPs and PVDF-based mesh were gathered using a 3D profiler (NanoView Inc., Daejeon, Korea) to determine the width and thickness.

## Results and discussion

An increase in the specific surface area of micro/nanostructured piezoelectric materials enhances the output performance of PENGs owing to an increase in the trapping region for charge transfer^26^. To increase the specific surface area of the piezoelectric PVDF mesh, we employed the EHD jet printing technique, allowing a higher printing resolution compared to the conventional inkjet printing techniques that operate with a thermal or piezoelectric printing module^27^. By applying an electric field, ink droplets are propelled from the nozzle onto a target substrate in the EHD printing mode, enabling the formation of a high-resolution Taylor cone jet down to the micrometer scale or lower. PVDF-based mesh patterns with a width of 13 μm and thickness of 1.54 μm [Fig. S2(a)] were achieved. For the PVDF-based mesh patterns, AgNP-based grid electrodes with a width of 18 μm and thickness of 1.19 μm [Fig. S2(b)] were constructed using the EHD printing technique.

PVDF can have α-, β-, γ-, and δ-phases^28^, among which the β-phase is desirable for PENG applications owing to its spontaneous polarization behavior^29^. However, PVDF tends to crystallize into the α-phase rather than the β-phase^30–32^. Hence, additional processes to change the α-phase to the β-phase are generally required. Nevertheless, according to Ye et al., the EHD jet-printed PVDF mesh exhibits a dominant β-phase^33^. Moreover, the FTIR spectra analysis shown in Fig. S3 confirms that the β-phase was dominant in the printed piezoelectric PVDF. Therefore, we did not conduct any additional processes to change the α-phase to the β-phase.

Vortex shedding occurs when fluid blows past a bluff body, producing a sinusoidal force. This is the origin of the vibration of the flexible sheet-type PENGs. If the vortex shedding frequency approaches the frequencies of the PENG structure, then flapping or chaotic flapping motion of the PENG, which is desirable for powering, can occur. In the case of a two-dimensional sheet with a high longitudinal stiffness and low bending stiffness, the structural restoring force of the sheet is governed by flow-induced tension^10^. Accordingly, the sheet's dynamic effect, surface vortex formation, wake vortex propagation, structural inertia, and changing force of the main body due to bending stiffness must be considered to predict the sheet’s mechanical response to vortex shedding. Therefore, *Re*, *K*_*B*_, and M* were regarded as the key parameters governing the mechanical response of the sheet^10^. *Re*, *K*_*B*_, and M* are defined as follows:

$$Re= \frac{{\rho }_{f}uL}{{\mu }_{f}},\quad {K}_{B}=\frac{E{h}^{3}/12(1-{v}^{2})}{{\rho }_{f}{u}^{2}{L}^{3}},\quad{M}^{*}=\frac{{\rho }_{s}h}{{\rho }_{f}L}$$

where *E*, $v$, $h$, ${\rho }_{f}, {\rho }_{s}, u$, *L*, and ${\mu }_{f}$ denote the Young’s modulus (MPa), Poisson’s ratio, thickness (m), fluid density (kg/m^3^), material density (kg/m^3^), flow velocity (m/s), two-dimensional body of length (m), and fluid viscosity (kg/m s), respectively. According to Lumpkin et al., the global distribution of the flow velocity in the Pacific, Atlantic, and Indian Oceans ranges from 0.031 to 0.656 m/s, and the corresponding *Re* values range from 6200 to 131,200^34^. We set the *Re* range from 1 to 141,489 in the present water channel experiments to simulate the ocean currents.

**Figure 2:**
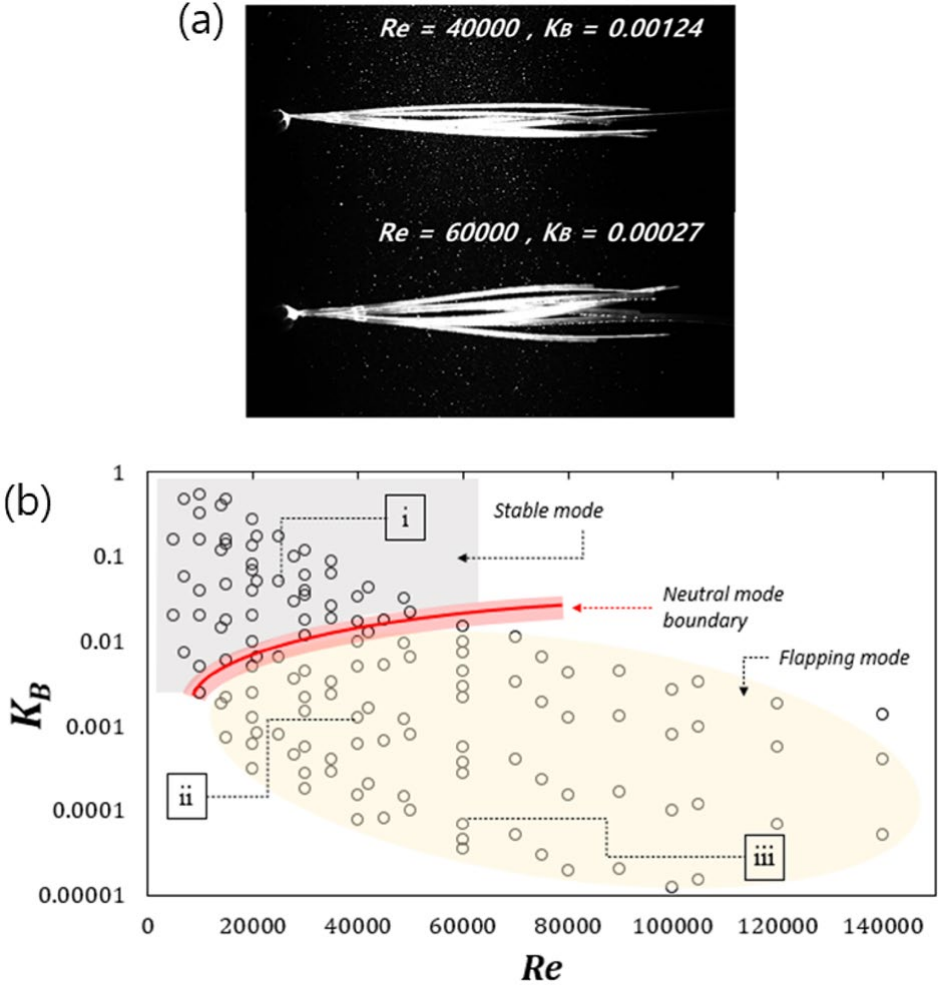
(**a**) Flapping sequence images of PENG deformation at each condition captured with a high-speed camera. (**b**) Regime map representing the PENG responses induced by the vortex shedding.

Figure 2a presents the flapping responses of the PENG at *Re* = 40,000 when *K*_*B*_ = 0.00124 and M* = 0.01, and at *Re* = 60,000 when *K*_*B*_ = 0.000046 and M* = 0.003, demonstrating that the flapping and chaotic flapping motion can be achieved in the water channel. Figure S4 demonstrates the response regime map as a function of M* versus *Re*, demonstrating stable and flapping modes. The initial neutral mode was found at M* = 0.005 and *Re* = 10,000, and the boundary was distributed at a higher M* as *Re* increased. Figure 2b presents the neutral curves between the stable (i) and flapping motions (ii and iii), demonstrating that a *K*_*B*_ value below 0.0024 can flap regardless of the *Re* range. Gurugubelli et al. reported that the neutral curve for a conventional foil appeared at a *K*_*B*_ value of approximately 0.001 at *Re* < 5000, which is in good agreement with our observations^35^. In addition, the regime in which the flapping motion is dominant can be divided into flapping (ii) and chaotic flapping (iii) regimes.

**Figure 3:**
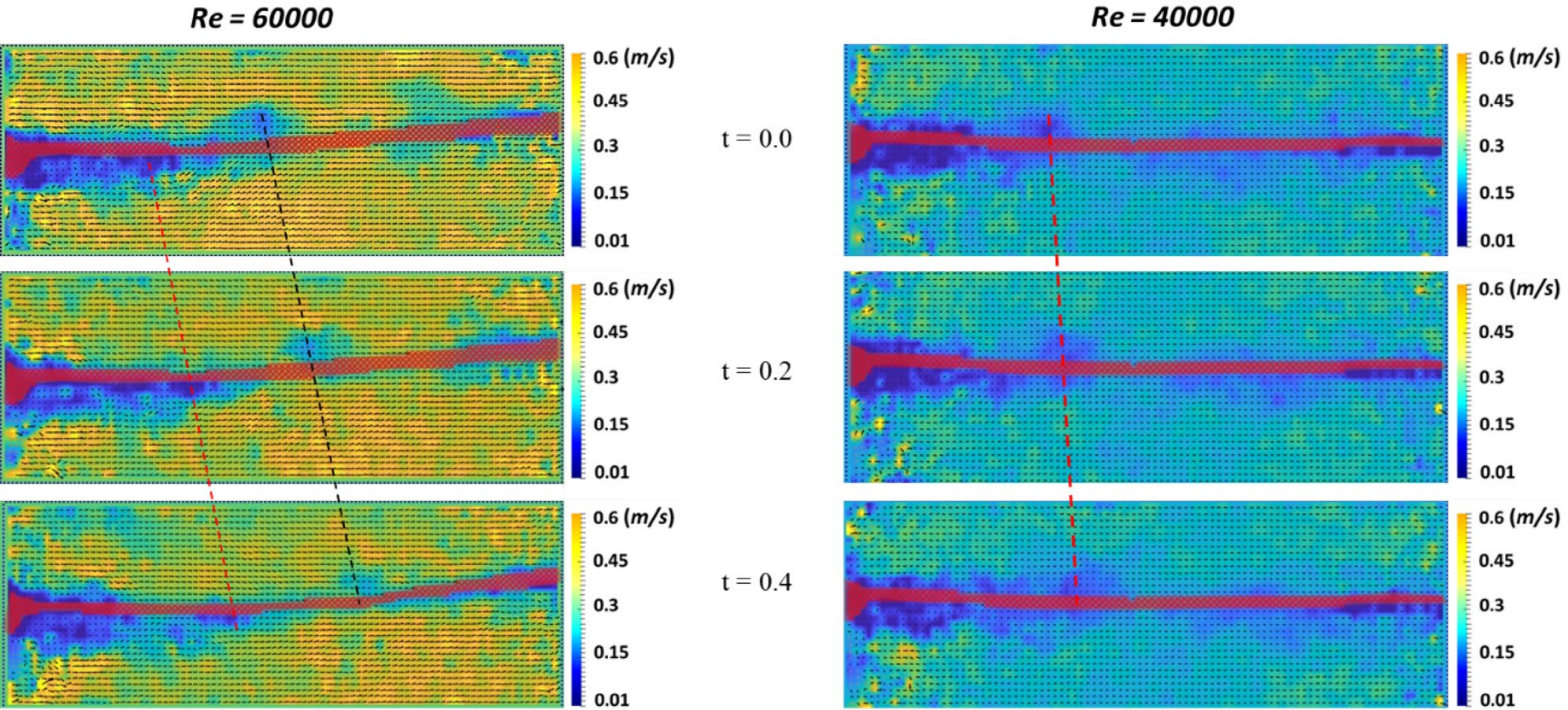
Instantaneous vortex contours and velocity vectors at *Re* = 60,000 and 4000, and t = 0, 0.2, 0.4.

As shown in Fig. 3, PIV was used to represent the vortex distribution and component velocity at t = 0, 0.2, and 0.4 s under flapping (*Re* = 40,000, *K*_*B*_ = 0.00124, M* = 0.01) and chaotic flapping regimes (*Re* = 60,000, *K*_*B*_ = 0.000046, M* = 0.003). Regardless of the PENG response, the flow velocities of the vortexes exhibited a comparable level of 0.013 m/s. However, in the case of the chaotic flapping regime, there were significant differences in the flow velocities between the vortex and surroundings, resulting in a pressure gradient along the y-axis. In contrast, the difference in the flow velocities was insignificant in the flapping regime. The sharp pressure gradient owing to the significant difference in the flow speed can be attributed to the larger displacement of the PENG in the chaotic flapping regime than that in the flapping regime.

**Figure 4:**
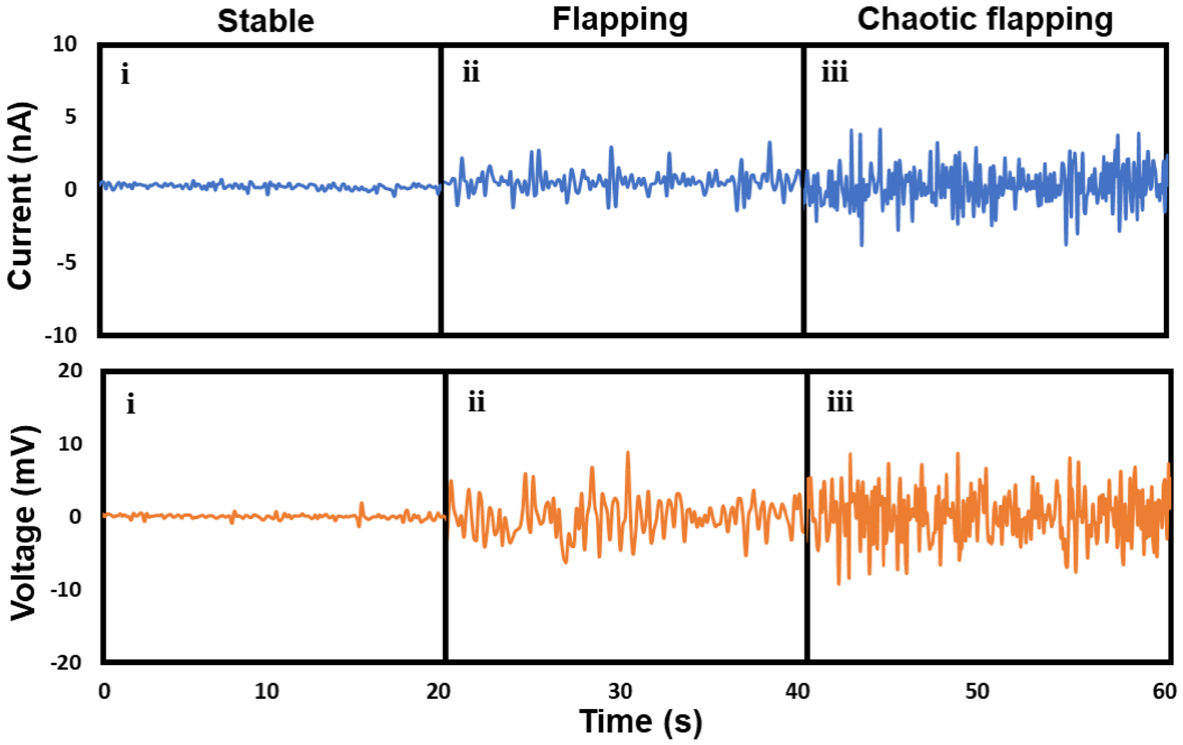
Output current and voltage measured under stable, flapping, and chaotic flapping regimes.

Figure 4 presents the output voltages and currents generated from the PENG under the stable, flapping, and chaotic flapping regimes. A schematic model of PENG interaction with water flow is displayed in Fig. S5(a). A schematic illustration of measurement circuit and electron flows depending on the PENG behaviors are given in Fig. S5(b) and (c). The acquired currents are sinusoidal due to the repetitive bending of the PENGs as displayed in Fig. S5(d).

As expected, Fig. 4 demonstrates that there was no output voltage or current in the stable regime owing to the absence of vibrations. In the flapping and chaotic flapping regimes, the PENG can generate electricity. The outputs generated in the chaotic flapping regime (17 mV and 8 nA) were greater than those in the flapping regime (9 mV and 4 nA), indicating that the PENG harvesting ocean current energy needs to be designed to exhibit a chaotic flapping response from the perspective of powering.

**Figure 5:**
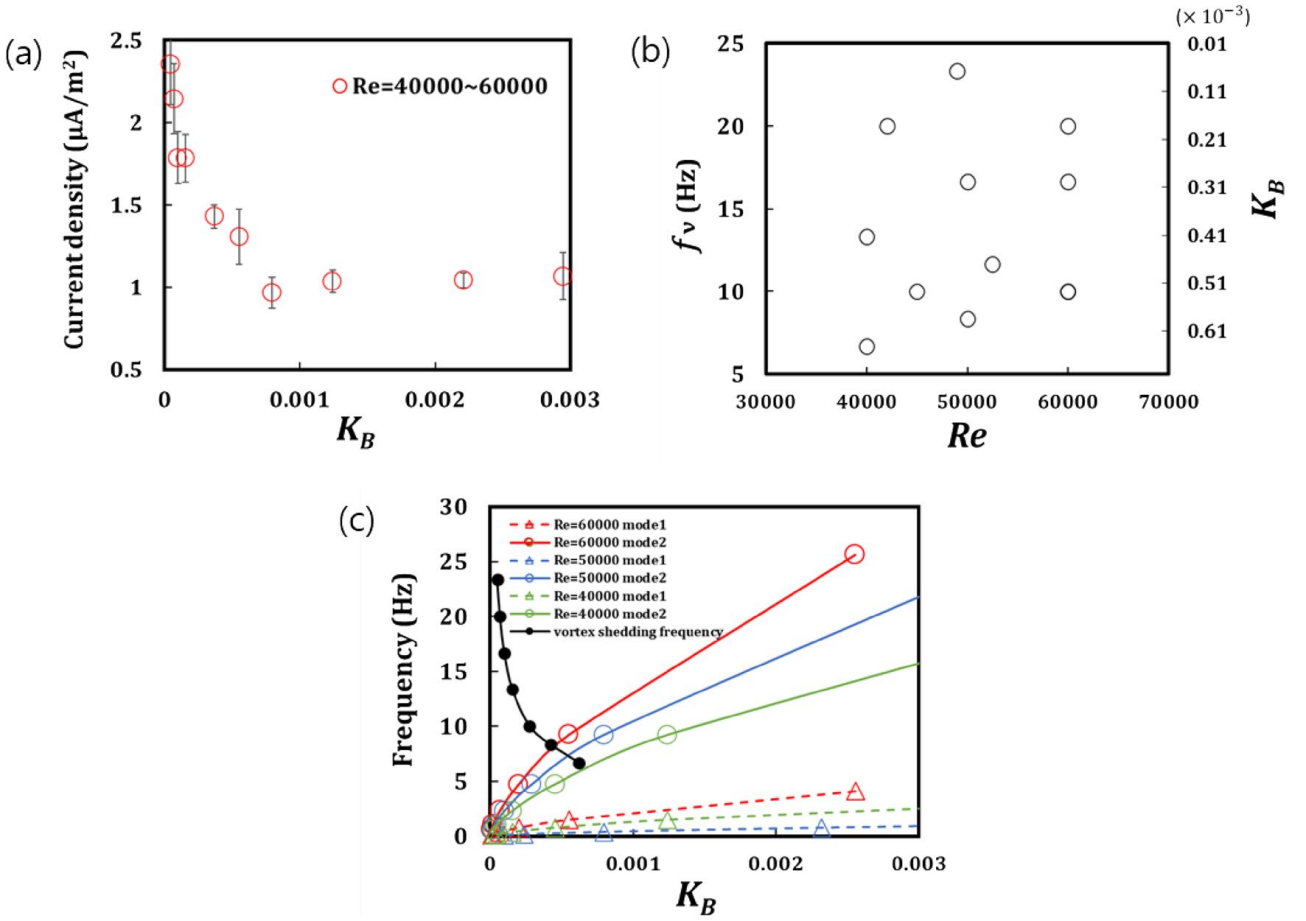
(**a**) Plot of the current density as a function of *K*_*B*_ measured in the *Re* range of 40,000 to 60,000. (**b**) Vortex shedding frequency and *K*_*B*_ as a function of *Re*. (**c**) Natural frequency of PENG and the vortex shedding frequency as a function of *K*_*B*_.

Figure 5a presents a plot of the current density as a function of *K*_*B*_ measured in the *Re* range of 40,000–60,000, which was chosen because it covers the flapping and chaotic flapping regimes with a change in the *K*_*B*_. A sharp increase from approximately 0.889 to 2.354 $\upmu$A/m^2^ was observed in the current density below a *K*_*B*_ value of 0.001, which can be attributed to both the resonance between the vortex shedding and the increase in the displacement of the PENG owing to the increase in its compliance. To test our hypothesis, we plotted the vortex shedding frequency and *K*_*B*_ value as a function of *Re* (Fig. 5b), and the natural frequency of the PENG and the vortex shedding frequency as a function of *K*_*B*_ (Fig. 5c). With *Re* ranging from 40,000 to 60,000, Fig. 5b demonstrates that the vortex shedding frequency and *K*_*B*_ values are distributed in the ranges of 5–25 and 0.000051–0.00062, respectively. Figure 5c demonstrates that the natural frequency of PENG mode 2 approached the vortex shedding frequency as *K*_*B*_ approached 0.00052. In contrast, the mode 1 frequency did not approach the vortex shedding frequency, indicating that the mode 2 resonance occurred more dominantly compared to mode 1. This analysis is in good agreement with the PIV image displayed in Fig. 2a, which presents mode 2 flapping. The current density sharply increased (Fig. 5a) below a *K*_*B*_ value of 0.00042, although the natural frequency of the PENG and the vortex shedding frequency receded into the distance. As previously indicated, we assumed that the large displacement of the PENG with a low *K*_*B*_ value may have led to the origin of the sharp increase in the power_._ The frequency of the output voltage in the chaotic flapping region displayed in Fig. 4 is approximately 4.33 Hz, which does not match the vortex shedding frequency (> 6.67 Hz). This indicates that the resonance between the vortex shedding and PENG is not the origin of the sharp increase in the current density. Therefore, the large displacement of the compliant PENG with a *K*_*B*_ below 0.00042 is the major contributor to the high current. Our observations indicate that the flexible sheet-type PENG should be designed to have a *K*_*B*_ value below the critical value for exhibiting chaotic flapping and generating significant power.

## Conclusion

Considering ocean conditions, we set a water channel system generating water flow with *Re* values ranging from 1 to 141,489. By plotting M* and *K*_*B*_ as a function of *Re*, we can draw neutral curves by dividing the stable and flapping regions. Depending on *Re*, it was found that the threshold values of M* and K_B_ exhibited a flapping motion. In particular, the sheet-type PENG with a *K*_*B*_ below 0.0024 can flap regardless of the *Re* range. We also observed that the PENG can generate significant power when it operates under a chaotic flapping regime. Using PIV, we observed rapid changes in the flow velocities around the PENG, causing a significant pressure gradient. We conclude that the significant pressure gradient is the origin of the large displacement of the PENG and efficient powering. In particular, in the *Re* range of 40,000 to 60,000, the current density of the PENG with *K*_*B*_ below 0.001 sharply increased as *K*_*B*_ decreased. By comparing the natural frequency of the PENG, vortex shedding frequency, and *K*_*B*_, we concluded that the sharp increase in the current density below a *K*_*B*_ value of 0.001 can be attributed to both the resonance between the vortex shedding and the increase in the displacement of the PENG owing to the increase in its compliance. From the perspective of efficient powering, we propose that the sheet-type PENG should be designed to have a *K*_*B*_ below the critical value under specific M* and *Re* conditions. Future studies should aim to replicate our findings under the real ocean condition. The reason is that the salinity, the pressure, etc. can affect the performance and the reliability of the PENGs. This is an important key component in future attempts to design effective and reliable PENGs with a *K*_*B*_ below the critical value under the real ocean condition.

## Supplementary Information

Supplementary Information.

## Data Availability

The data supporting the findings of this study are available from the corresponding author upon reasonable request.
