# Supplementary material for: Flapping and powering characteristics of a flexible piezoelectric nanogenerator at Reynolds number range simulating ocean current
Source: Sci Rep. 2022 Oct 1;12:16465. doi: 10.1038/s41598-022-20836-x (PMC9526697; doi:10.1038/s41598-022-20836-x)
Supplement: Supplementary file 1 — Supplementary Information. [file 41598_2022_20836_MOESM1_ESM.docx]

**Supplementary Information**

Flapping and powering characteristics of a flexible piezoelectric nanogenerator at Reynolds number range simulating ocean current

***Joonkyeong Moon^1^, Giho Kang^1^, Busi Im^1^, Jihoon Kim^2^, Dae-Hyun Cho^3,4,a)^, Doyoung Byun^1,a)^***

^1^Department of Mechanical Engineering, Sungkyunkwan University, Suwon 16419, Republic of Korea

^2^Coastal & Ocean Engineering Division, Korea Institute of Ocean Science and Technology, 385 Haeyang-ro, Busan Metropolitan City 49111

^3^Department of Mechatronics Engineering, Gyeongsang National University, Jinju 52725, Republic of Korea

^4^Department of Energy System Engineering, Gyeongsang National University, 33 Dongjin-ro, Jinju, Gyeongnam 52828, Republic of Korea

a)Authors to whom correspondence should be addressed: cho@gnu.ac.kr and [dybyun@skku.edu](mailto:dybyun@skku.edu)

Table S1. Summary of VIV-based PENGs

| **Ref** | **Materials** | **Source of vibration** | **Objective** | **Theoretical /**  **Experimental** | **Environment** | **Reynolds number**  **ranges** |
| --- | --- | --- | --- | --- | --- | --- |
| 4 | PVDF | Fluid-structure interaction by vortices generated from the wake of a rectangular cylinder | Optimization of the plate-wake interaction by controlling the diameter and thickness of a cylinder and the length of a plate | Experimental | water | < 40000 |
| 12 | PVDF | The resonance between PVDF-based PENG and vortex shedding | Finding a relationship between flow velocity, cylinder diameter, and the force applied to a beam. | Numerical /  Experimental | water | 2000 - 30000 |
| 14 | PVDF | An inverted flag flapping unstably in fluid flow | Analysis of the relation between flapping kinematics and performance considering bending rigidity and the inclination angle. | Numerical | x | < 400 |
| 18 | MFC/PVC | Vortex-induced vibration of two PENG beams from two cylinders | A novel energy harvesting system comprised of two PENG beams and two cylinders | Experimental | water | < 40000 |
| 32 | PVDF | Vibrations originated from turbulences by plates in a closed system flow channel | Generating electricity by placing turbulent plates in a closed flow channel and increasing turbulence intensity. | Numerical /  Experimental | water | 30825 |
| 33 | Piezo material | Vortex-induced pressure driving the plate to squeeze piezo-patches | Analysis of the effect of plate length and flow velocity on wake structure, and the effect of driving force on the performance | Numerical | x | 50000 - 90000 |
| 34 | Piezo material | Vortex shedding induced by various cylinders. | Output voltage measurement considering the vortex shedding frequency governed by various cylinders | Experimental | water | 350 - 1650 |
| 35 | PZT | Floating buoys on the water stream | Proposal of mass-spring model energy harvesting system using wave energy | Experimental | water | - |
| This  Work | PVDF | Vortex shedding from the cylinder and flow-induced vibration | Fluid-structure interaction study in a view of PENG powering under a wide range of *Re* for simulating real ocean | Experimental | water | 1 - 141489 |

*_
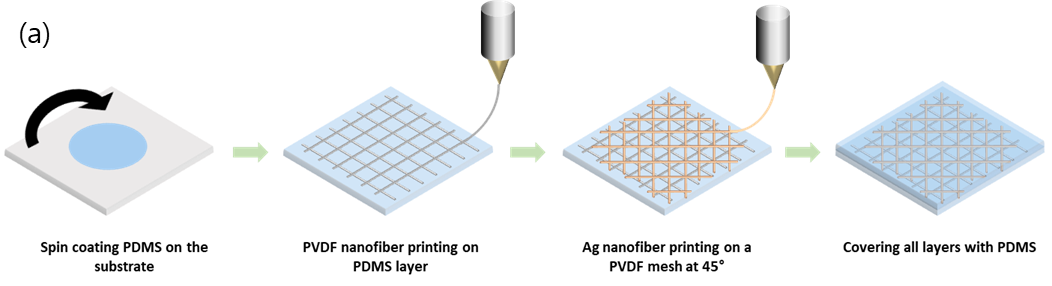
_*


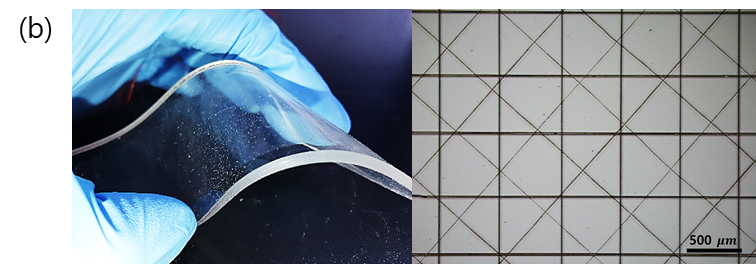


Fig. S1. (a) Schematic diagram of the PENG manufacturing process using EHD printing. (b) Optical images of the fabricated PENG.


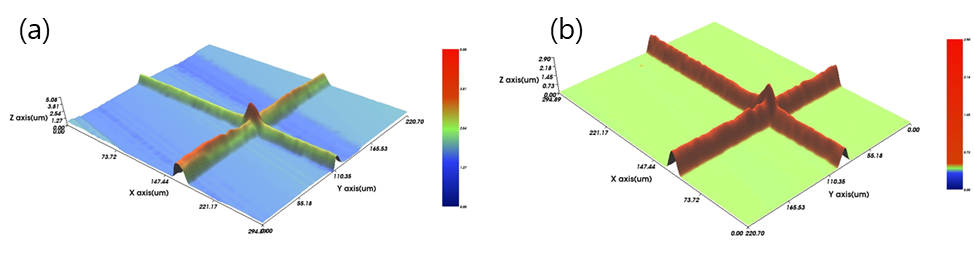


Fig. S2. 3D profile image of printed (a) PVDF-based and (b) AgNPs mesh.


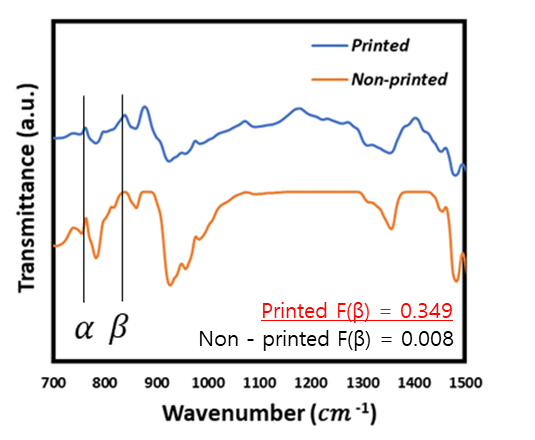


Fig. S3. FTIR spectra of EHD printed and non-printed PVDF-based nanofiber.


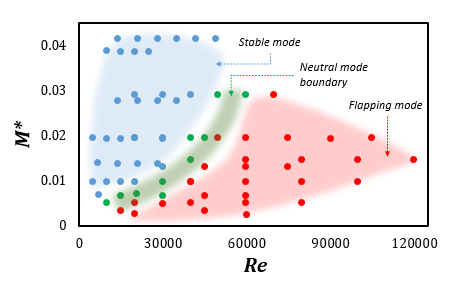


Fig. S4. Plot of *M** versus *Re* representing the flapping regime map.


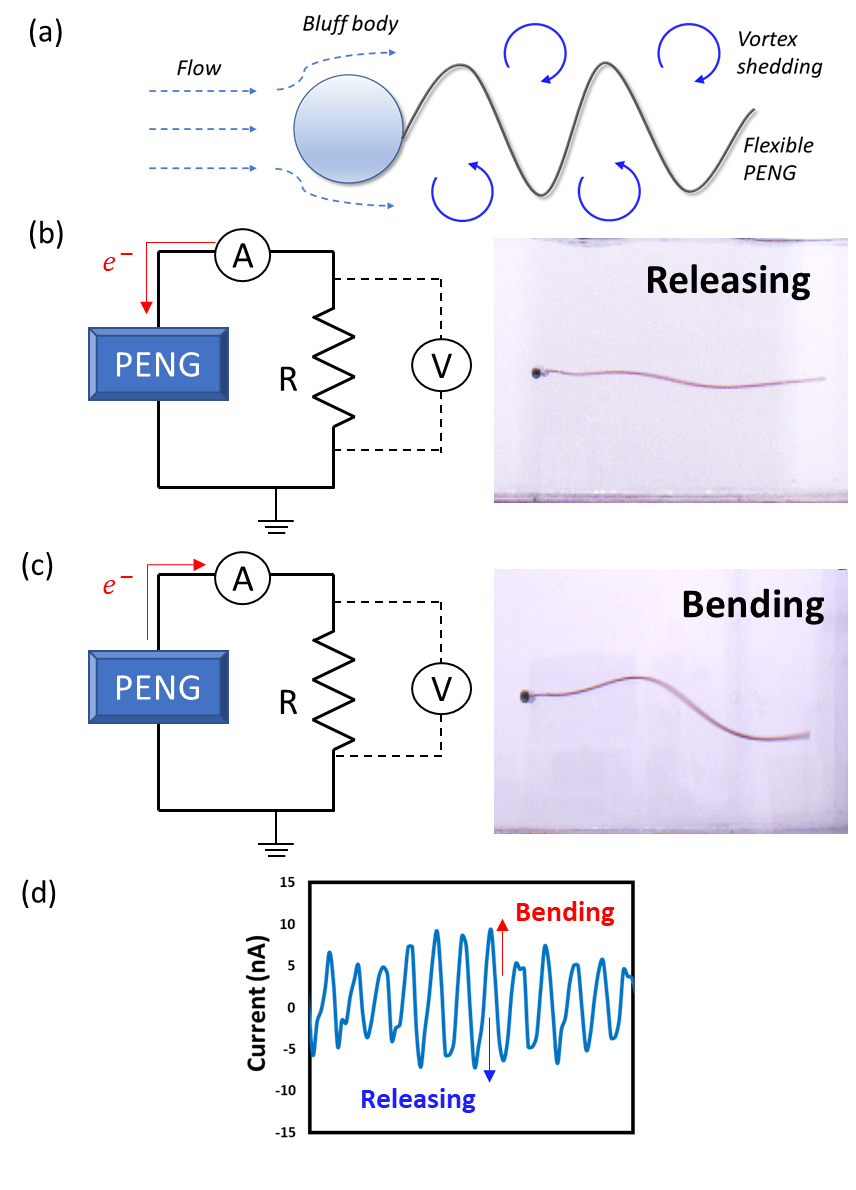


Fig. S5. Schematic model of PENG interaction with water flow (a). Schematic illustration of the measurement circuit and electron flows depending on the PENG behaviors (b and c). Measured currents as function of time (d). The resistance used in the measurements was 100MΩ.
